# STDP in lateral connections creates category-based perceptual cycles for invariance learning with multiple stimuli

**DOI:** 10.1007/s00422-014-0637-z

**Published:** 2014-12-09

**Authors:** Benjamin D. Evans, Simon M. Stringer

**Affiliations:** Oxford Centre for Theoretical Neuroscience and Artificial Intelligence, Department of Experimental Psychology, University of Oxford, Oxford, UK

**Keywords:** Transformation-invariant representations, Visual object recognition, Scene segmentation, Lateral plasticity, Spiking neural net, STDP

## Abstract

Learning to recognise objects and faces is an important and challenging problem tackled by the primate ventral visual system. One major difficulty lies in recognising an object despite profound differences in the retinal images it projects, due to changes in view, scale, position and other identity-preserving transformations. Several models of the ventral visual system have been successful in coping with these issues, but have typically been privileged by exposure to only one object at a time. In natural scenes, however, the challenges of object recognition are typically further compounded by the presence of several objects which should be perceived as distinct entities. In the present work, we explore one possible mechanism by which the visual system may overcome these two difficulties simultaneously, through segmenting unseen (artificial) stimuli using information about their category encoded in plastic lateral connections. We demonstrate that these experience-guided lateral interactions robustly organise input representations into perceptual cycles, allowing feed-forward connections trained with spike-timing-dependent plasticity to form independent, translation-invariant output representations. We present these simulations as a functional explanation for the role of plasticity in the lateral connectivity of visual cortex.

## Introduction

In our natural visual experience, objects are rarely seen in isolation. In order to make sense of the visual world, the brain must differentiate each object from others in the field of view. Once the objects in a scene have been reliably segmented, downstream neurons may begin to form transformation-invariant representations of each independently. How the visual system begins to distinguish one object from another is still the subject of speculation, however. The aim of the present study is to explore how visual experience may lead to an automatic, unsupervised mechanism to aid in this process.

Through studying the structure of the primate ventral visual stream (Felleman and Van Essen 1991) and the progressive changes in its cell response properties (Kobatake and Tanaka 1994; Tanaka 1996; Connor et al. 2007), neurophysiologists and theorists have converged upon the idea of a hierarchical processing system (DiCarlo et al. 2012). Tolerance to image transformations is gradually increased through changes to the neuronal representations found at each layer, while information about specific identities is learnt and encoded in the synapses between neurons in successive layers. Learning mechanisms which utilise the statistics of natural scenes are believed to facilitate this process by making the representations tolerant to identity-preserving transformations (DiCarlo and Cox 2007; DiCarlo et al. 2012). Trace (Földiák 1991) and CT learning (Stringer et al. 2006) help to associate different images with temporal (Wallis and Rolls 1997; Li and DiCarlo 2008) and spatial overlap (Stringer et al. 2006), respectively, which are likely to represent the same objects.

The increasingly large receptive field sizes along the ventral visual stream (Freeman and Simoncelli 2011) help to build cell responses with tolerance to stimulus size (Ito et al. 1995; Hung et al. 2005) and position (Tovée et al. 1994; Op de Beeck and Vogels 2000; Hung et al. 2005), but at the cost of failing to preserve spatial information. This is not a problem when only one object is in the field of view, but poses a significant challenge for the visual system when it experiences more natural scenes composed of multiple objects. The challenge lies in combining the features of one stimulus into a coherent percept while simultaneously segmenting them from the features of the other stimulus, an issue known as the ‘binding problem’ (Rousselet et al. 2004).

With simultaneously active visual representations, the learning mechanisms which help to associate together different transforms of the same object can also be counter-productive when several objects are presented in the same scene. Under such circumstances, simultaneously presented objects (or some particular views of them) will tend to be combined into the same output representation, leading to the ‘superposition catastrophe’ (von der Malsburg 1999). Even if the resultant neural representations have developed some ability to generalise (i.e. become tolerant to identity-preserving transformations), they will be unable to discriminate between these different objects, limiting the recognition abilities of the system.

As a consequence, previous attempts to model the learning processes of the ventral visual system have been limited by the need to present stimuli individually during training (Fukushima 1980, 1988; Wallis and Rolls 1997; Perry et al. 2006; Stringer et al. 2006) in order to avoid the superposition catastrophe, including feed-forward models learning visual category features through STDP (Masquelier and Thorpe 2007). While this single stimulus presentation has been a useful first step in understanding how temporal and spatial continuity can lead to view-invariant representations, several researchers have recognised the paradigm’s lack of ecological validity since natural visual scenes typically involve multiple stimuli (Stringer et al. 2007; Stringer and Rolls 2008). Not only is this paradigm limiting for real-world applications and convincing models of the visual system, but there is even evidence that the learning abilities of such networks may be enhanced by the simultaneous presence of multiple objects rather than stimuli presented in isolation (Spratling 2005).

Several mechanisms have been proposed by which these models (and the brain) may disentangle such combined representations. One such proposition is that an attentional spotlight means that we only perceive one object at a time, even though multiple objects may be present in our visual fields (Rolls and Deco 2002). Such an attentional mechanism has been successfully implemented in a spiking model, whereby the firing threshold was reduced in neurons corresponding to the attended area (VanRullen and Thorpe 2002). However, this begs the question of how the objects are segmented in the first instance (in order for attention to select one out of the entire scene) and so requires further investigation.

Other plausible solutions to the problem have come from considering not just the properties of the model, but also the statistics of the environment. By presenting different combinations of stimuli to a simple one-layer competitive neural network, it is able to separate out the stimuli and form individual representations despite only being trained on multiple objects (Stringer and Rolls 2008). This process requires a sufficient degree of ‘statistical decoupling’ between objects, however (i.e. each object is experienced with a sufficient number of other objects on separate occasions), which may not always be available to the observer.

Progressing this research, it was found that another mechanism requiring less extensive training was to show the network pairs of objects moving independently, for example, rotating at different speeds or in different directions (Tromans et al. 2012). Again, this relies upon a more realistic training environment rather than any additional properties of the model and it seems likely that there are other mechanisms in an ecologically valid visual scene for helping to segment and learn about objects independently.

Research conducted on these mechanisms has so far largely been with rate-coded neural network models. However, theoretical analysis has shown how assemblies of *spiking* neurons may either synchronise or desynchronise depending upon the nature of their lateral interactions (Nischwitz and Glünder 1995). According to the ‘binding-by-synchrony’ hypothesis (Milner 1974; Engel et al. 1991), such synchronised neural assemblies form a coherent stimulus percept, with psychophysics studies suggesting they are perceived as distinct from other assemblies with phase-shifted firing (Usher and Donnelly 1998). Modelling the individual action potentials of neurons may therefore provide a mechanism beyond the scope of rate-coded models to both overcome the binding problem through ‘feature-linking’ (Gray et al. 1989) and simultaneously segment different stimuli within the same scene.

Previously, we demonstrated how biologically plausible properties of a *spiking* neural network may provide additional ways to overcome the superposition catastrophe in this way. This involved desynchronising the volleys of spikes representing each stimulus with respect to each other through hard-wired lateral connections and cell firing-rate adaptation in a competitive network (Evans and Stringer 2013). This ‘Mexican hat’ lateral connectivity profile (consisting of short-range excitation with long-range/global inhibition) proved to be an effective mechanism for desynchronising spatially separate representations. However, different objects may at times be brought close together, and even overlap in the field of view, yet still be recognised as independent objects.

This paper explores the effects of early exposure to stimuli in building psychological categories of objects to aid the process of scene segmentation. The novel step in this paper, compared to the model of Evans and Stringer (2013), is to introduce plasticity into the lateral excitatory–excitatory ($ElE$) connections of the first neuronal layer. Rather than associating the common features of a category of stimuli in the feed-forward synapses between layers, the prototypical features may instead be grouped through a strengthening of the lateral connections between them. If two stimuli are from sufficiently dissimilar categories, the membership information encoded in the lateral connections should push the representations out of phase in a more precise way, even when the stimuli are close or partially overlapping on the retina. Furthermore, this method of automatic scene segmentation should then enhance learning in downstream areas of the brain as previously suggested (Miconi and VanRullen 2010), allowing transformation-invariant representations of multiple stimuli to be formed simultaneously.

### Overview of the model

In the present paper, learning is implemented in the lateral excitatory connections through an implementation of STDP (Perrinet et al. 2001) rather than hard-wiring a ‘Mexican hat’ architecture. It is then investigated whether a single layer of laterally connected excitatory and inhibitory neurons is able to use these lateral connections to learn about the visual categories from examples it is presented with. Idealised but artificial stimuli were chosen for these simulations to simplify the model and make its mechanism of operation more apparent. After an initial phase of encoding category information in the lateral connections, the network is then able to segment simultaneously presented novel stimuli through organising their input representations into anti-phase oscillations. It is then shown how this dynamic of ‘perceptual cycles’ (Miconi and VanRullen 2010) may be used to learn transformation-invariant representations in the output layer. This is achieved through modification of the feed-forward excitatory connections by the continuous transformation (CT) Learning mechanism (Stringer et al. 2006) as the stimuli translate across the input layer.

The first section of research in this paper (Sect. 3.1) explores how the preliminary learning of category information in the excitatory lateral connections allows the network to push representations of two previously unseen stimuli from different categories out of phase with respect to one another (based upon the prior learning about their categories). This experience-dependent scene segmentation through perceptual cycles is explored with respect to variations in several key parameters.

During the initial training phase, the network was presented with ‘example stimuli’ from each of two categories where the examples were composed of a fixed number of neurons (representing input ‘features’), drawn randomly from their respective prototype pools of neurons. The neurons of a particular example were then activated with an external current such that each example was presented to the network individually. Here, it is assumed that stimuli from the same category share a proportion of their features in common with each other and that members of different categories have far fewer features in common. In the idealised simulations of this work, there were typically no shared features between different categories, except in the simulations where a limited number of shared features were introduced specifically to investigate overlap between categories.

As the example stimuli are presented to the network, the coactivity of the features of a category leads to their association through the spike-timing-dependent plasticity (STDP) learning rule in the excitatory lateral connections. This first stage serves to strengthen these initially weak connections between neurons representing features of the same category, particularly strengthening connections between the most prototypical features of a category.

After building up the lateral connections in this way, the network is then tested by presenting a combination of two *novel* examples, representing a simple visual scene composed of two objects. It is hypothesised that the effect of training the lateral connections will be that the two stimuli are segmented through anti-phase oscillations. In particular, the neural firing representing the features of a particular example will be synchronised with respect to the other features of the same example (due to the strengthened lateral connections between features of the same category) and desynchronised with respect to the features of the other example (due to the weak connections between features of different categories).

The second section of research presented (Sect. 3.2) augments the network to incorporate a second layer of neurons with excitatory, plastic feed-forward connections between the pyramidal cells of each layer. The aim of this work is to investigate how such higher layers may exploit the input layer dynamics formed from prior learning about categories in order to segment a visual scene composed of multiple stimuli and learn transformation-invariant representations of them as they move in lockstep across the input layer.

A similar training paradigm to the first section is used in the prior training phase of this section, except that the input layer is extended and the individually presented stimuli translate across it. This allows the network to build strong lateral connections between neurons representing the different transforms (that is, locations) of stimuli in the same category, without strengthening connections between transforms of different categories.

During the second phase of training, the lateral excitatory synaptic weights are prevented from further modification and learning commences in the excitatory feed-forward connections utilising the same STDP learning rule used to train the lateral connections in the prior phase. A pair of novel stimuli are presented together to the network in the same portion of the input layer, which are shifted progressively across the input layer in lockstep.

The hypothesis tested is that the anti-phase oscillations generated by the learning in the plasticity of the input layer’s lateral excitatory connections will allow the output neurons to learn to respond selectively to either one (but not both) of the two novel stimuli across most or all of their transforms. This should be possible due to the temporal specificity of the STDP learning rule in the feed-forward connections between successive layers, whereby significant LTP occurs only if the input spike precedes the output spike within a narrow time window. Due to the self-organised dynamics of the input layer representations in which two stimuli are out of phase, this should occur for one particular stimulus at a time. Conversely, the spike volley of the other stimulus should fall outside of this LTP time window, instead coming after the output spikes and subjecting the corresponding synapses to LTD. This should allow transformation-invariant representations to form individually for the translating stimuli through the CT learning mechanism, as explored in earlier work (Evans and Stringer 2012, 2013).

## Methods

### Model architecture

**Fig. 1:**
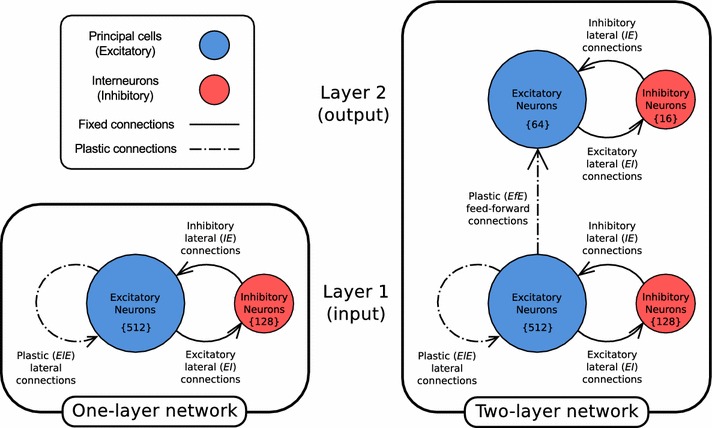
Network architecture for the one and two-layer networks. *Each layer* has a pool of excitatory (principal) neurons reciprocally connected to a pool of inhibitory interneurons. In the first layer, the excitatory neurons have recurrent plastic connections. In the two-layer model, a *second layer* is added to the network with plastic feed-forward connections between the excitatory neurons

The architectures of both networks used are illustrated in Fig. 1. In the first set of simulations described in Sect. 3.1, the network was a single layer of 512 conductance-based leaky integrate-and-fire (gLIF) excitatory pyramidal neurons interconnected with plastic lateral excitatory connections initialised to zero strength. Unlike the simulations of Evans and Stringer (2013), these lateral connections were modified through learning rather than imposing a fixed ‘Mexican Hat’ profile upon them. There was also a separate pool of 128 inhibitory interneurons with fixed strength lateral connections to and from each of the excitatory cells. The pyramidal cells featured cell firing-rate adaptation, mediated by calcium-gated potassium currents, and all synapses were conductance-based, as described in Sect. 2.2. Plastic synapses, both lateral ($ElE$) and feed-forward ($EfE$), featured an online, multiplicative form of STDP derived from Perrinet et al. (2001), as described in Sect. 2.4. General parameters for this model are given in Table 1.

In the second set of simulations with translating stimuli described in Sect. 3.2 with general parameters given in Table 2, the same basic architecture was used but incorporated an additional output layer of 64 excitatory pyramidal cells. Importantly, the addition of an output layer also meant that there were feed-forward excitatory connections ($EfE$) from pyramidal cells in the input layer to pyramidal cells in the output layer (as shown in Fig. 1). These feed-forward connections were modified during training using the same STDP learning rule used in the excitatory to excitatory lateral connections but initialised randomly and uniformly in the range $[0, \lambda \Delta g_{\max }]$. Each of the two layers of pyramidal cells had their own pool of inhibitory interneurons in a ratio of 4:1 ($E$:$I$). The input layer excitatory pyramidal neurons were arranged into a spatial configuration of 32 neurons deep by 16 neurons wide to accommodate translating stimuli (instead of being one-dimensional as before), while the output layer was arranged in an $8 \times 8$ configuration.

**Table 1:** Default parameters for one-layer network

Network parameters	Symbol	Value
Cue current	$I^\mathrm{ext}$	0.75 nA
Cue period {training, testing}	$t_\mathrm{cue}$	{500, 1,000} ms
Number of training epochs	$N_\mathrm{epochs}$	10
Time step for numerical integration	$\Delta t$	0.02 ms
Number of layers	$N_\mathrm{L}$	1
Number of excitatory cells per layer	$N_\mathrm{E}$	512
Number of inhibitory cells per layer	$N_\mathrm{I}$	128
Prob. of $E$ cell synapsing with afferent lateral $E$ cell	$p(ElE)$	$0.5$
Prob. of $E$ cell synapsing with afferent $I$ cell	$p(IE)$	$1.0$
Prob. of $I$ cell synapsing with afferent $E$ cell	$p(EI)$	$1.0$
Prob. of $I$ cell synapsing with afferent $I$ cell	$p(II)$	$0.0$

Sets of values are indicated by braces whereby the values correspond to the parameters used for the training/testing periods

**Table 2:** Default parameters for two-layer network

Network parameters	Symbol	Value
Cue current	$I^\mathrm{ext}$	0.75 nA
Cue period {training, testing}	$t_\mathrm{cue}$	{500, 1,000} ms
Number of training epochs	$N_\mathrm{epochs}$	10
Time step for numerical integration	$\Delta t$	0.02 ms
Number of layers	$N_\mathrm{L}$	2
Number of excitatory cells per layer	$N_\mathrm{E}$	{512, 64}
Number of inhibitory cells per layer	$N_\mathrm{I}$	{128, 16}
Prob. of $E$ cell synapsing with afferent feed-forward $E$ cell	$p(EfE)$	$\{-, 1.0\}$
Prob. of $E$ cell synapsing with afferent lateral $E$ cell	$p(ElE)$	$\{1.0, 0.0\}$
Prob. of $E$ cell synapsing with afferent $I$ cell	$p(IE)$	$\{1.0, 1.0\}$
Prob. of $I$ cell synapsing with afferent $E$ cell	$p(EI)$	$\{1.0, 1.0\}$
Prob. of $I$ cell synapsing with afferent $I$ cell	$p(II)$	$\{0.0, 0.0\}$

Sets of values are indicated by braces whereby the values correspond to the parameters used for each of the two layers of neurons (or the training/testing periods in the case of the cue period)

### Neuron model description

The neurons used in this work are conductance-based leaky integrate-and-fire neurons (gLIF) including excitatory and inhibitory classes (indexed by $\gamma$), with zero-mean Gaussian white noise added to the cell membrane potential. Here, the standard deviation $\sigma ^\gamma = 0.015\cdot \left( \varTheta ^\gamma - V_\mathrm{H}^\gamma \right)$ (1.5 % of the difference between the firing threshold and the hyperpolarisation potential) as used by Masquelier et al. (2009) and $\xi (t)$ is a Wiener (Gaussian) variable (representing $\frac{\hbox {d}W}{\hbox {d}t}$ and satisfying the definition of the Wiener process). The membrane potential model also incorporates cell firing-rate adaptation through calcium-gated potassium channels. The cellular parameters used were the default values described in Table 3 from Troyer et al. (1998) and the default STDP parameters used are as detailed in Table 4 (Troyer et al. 1998; Perrinet et al. 2001).

**Table 3:** Cellular parameters

Cellular parameters	Symbol	Value
Excitatory cell somatic capacitance	$C_{m}^\mathrm{E}$	500 pF
Inhibitory cell somatic capacitance	$C_{m}^\mathrm{I}$	214 pF
Excitatory cell somatic leakage conductance	$g_{0}^\mathrm{E}$	25 nS
Inhibitory cell somatic leakage conductance	$g_{0}^\mathrm{I}$	18 nS
Excitatory cell resting potential	$E_\mathrm{L}^\mathrm{E}$	$-$74 mV
Inhibitory cell resting potential	$E_\mathrm{L}^\mathrm{I}$	$-$82 mV
Excitatory firing threshold potential	$\varTheta ^\mathrm{E}$	$-$53 mV
Inhibitory firing threshold potential	$\varTheta ^\mathrm{I}$	$-$53 mV
Excitatory after-spike hyperpolarisation potential	$V_\mathrm{H}^\mathrm{E}$	$-$57 mV
Inhibitory after-spike hyperpolarisation potential	$V_\mathrm{H}^\mathrm{I}$	$-$58 mV
Absolute refractory period	$\tau _{R}$	2 ms
Increase in adaptation (potassium) conductance	$g_\mathrm{K}[\alpha ]$	[0.6, 60] nS
Potassium reversal potential	$E_\mathrm{K}$	$-$80 mV
Adaptation (calcium decay) time constant	$\tau _\mathrm{Ca}$	[5, 5,000] ms

The leaky integrate-and-fire parameters used by default throughout this paper were taken from Troyer et al. (1998) (derived originally from cortical electrophysiological recordings (McCormick et al. 1985) with the adaptation parameters mostly taken from Liu and Wang (2001). Square brackets are used to indicate ranges of parameters which were explored through simulations

The dynamics of the cell membrane potential $V_i(t)$ are governed by Eq. 1. Here, $g_0^\gamma$ is the standard leakage conductance, determining the core behaviour of gLIF neurons. Any difference between the present membrane potential and the resting potential for that class of neuron, $E_\mathrm{L}^\gamma$ results in a leakage current driving the membrane potential back towards the resting potential. This happens over a time course governed by the membrane time constant,  (20 and 12 ms for excitatory and inhibitory cells, respectively).

The excitatory neurons also feature an adaptation mechanism through a calcium-activated $\hbox {K}^+$ conductance, $g_\mathrm{K}\cdot [\hbox {Ca}^{2+}]$. Following recent spiking activity, calcium activates the potassium channels, increasing their conductance. The resultant adaptation currents flow down the gradient towards the potassium reversal potential, $E_\mathrm{K} = -80$ mV, decreasing the firing rate of the cell for a constant input.

1 $$\begin{aligned} C_m^\gamma \frac{\hbox {d}V_i(t)}{\hbox {d}t}&= g_0^\gamma \left( E_\mathrm{L}^\gamma - V_i(t)\right) + g_\mathrm{K}[\hbox {Ca}^{2+}]_i(E_\mathrm{K} - V_i(t)) \nonumber \\&+ \sum _{j \in \{E\}} g_{ij}(t) \left( E_\mathrm{syn}^\mathrm{E} - V_i(t)\right) \nonumber \\&+ \sum _{k \in \{I\}} g_{ik}(t) \left( E_\mathrm{syn}^\mathrm{I} - V_i(t)\right) \nonumber \\&+ I_{i}^\mathrm{ext}(t) + \sigma ^\gamma \xi (t) \sqrt{\tau _m} \end{aligned}$$

The arrival of spikes from presynaptic excitatory or inhibitory cells increases the conductances $g_{ij}(t)$ and $g_{ik}(t)$ at those synapses, driving the cell potential towards the excitatory ($E_\mathrm{syn}^\mathrm{E}$) or inhibitory ($E_\mathrm{syn}^\mathrm{I}$) synaptic reversal potential, respectively. Additionally, $I_{i}^\mathrm{ext}(t)$ models the directly injected current with which the neurons may be stimulated to represent the visual inputs.

Upon reaching its firing threshold potential $\varTheta ^\gamma$, a cell emits a single spike and its membrane potential is reset to its after-spike hyperpolarisation potential $V_\mathrm{H}^\gamma$. For excitatory cells, this also increases the cytoplasmic concentration of calcium $[\hbox {Ca}^{2+}]$ by $\alpha$ (such that typically $g_\mathrm{K}[\alpha ] = 6$ nS) as shown in Eq. 2. The time constant for the decay rate of $[\hbox {Ca}^{2+}]$ back to its initial value of $0$ is governed by the time constant $\tau _\mathrm{Ca} = 50$ ms.

2 $$\begin{aligned} \frac{\hbox {d}[\hbox {Ca}^{2+}]_i}{\hbox {d}t} = -\frac{[\hbox {Ca}^{2+}]_i}{\tau _\mathrm{Ca}} +\alpha \sum _q \delta \left( t - t_{i}^q\right) \end{aligned}$$

**Table 4:** Synaptic parameters

Synaptic parameters	Symbol	Value	
Synaptic neurotransmitter concentration	$\alpha _{C}$	0.5	†
Proportion of unblocked NMDA receptors	$\alpha _{D}$	0.5	†
Presynaptic STDP time constant	$\tau _\mathrm{C}$	15 ms	†
Postsynaptic STDP time constant	$\tau _\mathrm{D}$	25 ms	†
Synaptic learning rate	$\tau _{\Delta g}$	0.1 ms	†
Maximum change in $EfE$ synaptic conductance	$\lambda ^\mathrm{EfE}$	[0, 3.75] nS	*
Maximum change in $ElE$ synaptic conductance	$\lambda ^\mathrm{ElE}$	[0.05, 500] nS	*
Change in synaptic conductance ($I\rightarrow E$)	$\lambda ^\mathrm{IE}$	[0.05, 500] nS	*
Change in synaptic conductance ($E\rightarrow I$)	$\lambda ^\mathrm{EI}$	5.0 nS	*
Excitatory synapse reversal potential	$E_\mathrm{syn}^\mathrm{E}$	0 mV	§
Inhibitory synapse reversal potential	$E_\mathrm{syn}^\mathrm{I}$	$-$70 mV	§
Excitatory–excitatory synaptic time constant	$\tau _\mathrm{EE}$	1 ms	
Inhibitory–excitatory synaptic time constant	$\tau _\mathrm{IE}$	5 ms	§
Excitatory–inhibitory synaptic time constant	$\tau _\mathrm{EI}$	2 ms	§

The synaptic reversal potentials and conductance time constants were taken from the same studies as the cellular parameters (Troyer et al. 1998; McCormick et al. 1985) as indicated by §. Plasticity parameters (denoted by †) are taken from Perrinet et al. (2001). Parameters marked with * were tuned for the reported simulations, and ranges were systematically explored where indicated by square brackets

The dynamics governing the conductance of a particular synapse, $g_{ij}(t)$ (indexed by $ij$), are governed by a synaptic time constant ($\tau _g \in \{\tau _\mathrm{EE}, \tau _\mathrm{IE}, \tau _\mathrm{EI}\}$) and a Dirac delta function for when spikes occur (thus neglecting the shape of the action potential) as described in Eq. 3. Here, $\Delta g_{ij}$ is constrained to the range $[0,1]$ when plastic, or set to $1$ when fixed. Consequently, the coefficient $\lambda$ is introduced to scale the conductance increment to a biologically realistic value for each class of synapse, as detailed in Table 4.

3 $$\begin{aligned} \dfrac{\hbox {d}g_{ij}(t)}{\hbox {d}t} = -\dfrac{g_{ij}(t)}{\tau _g} + \lambda \Delta g_{ij}(t) \sum _q \delta \left( t - t_{j}^q\right) \end{aligned}$$

### Lateral connectivity

Excitatory neurons within each layer were connected to other excitatory neurons according to the probability $p(ElE) = 0.5$ (disallowing self-synapses). Their synaptic weights $\Delta g_{ij}^\mathrm{ElE}$ were initialised to zero and adjusted during the course of training according to the STDP learning rule described in Sect. 2.4 (Eqs. 4–6). The excitatory neurons were fully connected to the inhibitory neurons and vice-versa within each layer; however, unlike the $ElE$ (and $EfE$) synapses, $EI$ and $IE$ synapses were not plastic but instead initialised to $\lambda ^\mathrm{EI}\Delta g = \lambda ^\mathrm{IE}\Delta g = 5$ nS by default.

### Synaptic learning

To investigate the input dynamics upon learning, the excitatory–excitatory connections were modified by an online, multiplicative form (van Rossum et al. 2000; Gütig et al. 2003) of spike-timing-dependent plasticity formulated by Perrinet et al. (2001) and described in Eqs. 4–6. In contrast to additive forms of STDP, multiplicative forms yield a normal distribution of synaptic efficacies as has been found experimentally (Bi and Poo 1998) rather than creating two populations at the extremes (Song et al. 2000). Both the excitatory lateral connections ($ElE$) and (when present) the excitatory feed-forward connections ($EfE$) were modified through learning according to these rules, while all other connections to and from inhibitory interneurons ($IE$ and $EI$) were fixed throughout each simulation.

Each plastic synapse has a differential equation describing a plasticity variable $C_{ij}$ modelling a trace of recent presynaptic activity, which may be thought of as the concentration of glutamate released into the synaptic cleft (Perrinet et al. 2001). It is bounded by $[0,1]$ for $0 \leqslant \alpha _\mathrm{C} < 1$ and is described in Eq. 4, where $t_j^q$ is the time of the $q$th spike emitted by the $j$th presynaptic cell.

4 $$\begin{aligned} \dfrac{\hbox {d}C_{ij}(t)}{\hbox {d}t} = -\dfrac{C_{ij}(t)}{\tau _\mathrm{C}} + \alpha _\mathrm{C}\left( 1-C_{ij}(t)\right) \sum _q\delta \left( t-t_{j}^q\right) \end{aligned}$$

The presynaptic spikes drive $C_{ij}(t)$ up at a synapse according to the model parameter $\alpha _\mathrm{C}$ and the current value of $C_{ij}(t)$, which then decays back to 0 over a time course governed by $\tau _\mathrm{C}$.

The recent postsynaptic activity, $D_i(t)$, is modelled by Eq. 5, which may be interpreted as the proportion of unblocked NMDA receptors as a result of recent depolarisation through back-propagated action potentials (Perrinet et al. 2001). Here, $t_i^p$ is the time of the *p*th spike emitted by the *i*th postsynaptic cell.

5 $$\begin{aligned} \dfrac{\hbox {d}D_i(t)}{\hbox {d}t} = -\dfrac{D_i(t)}{\tau _\mathrm{D}} + \alpha _\mathrm{D}\left( 1-D_i(t)\right) \sum _p\delta \left( t-t_{i}^p\right) \end{aligned}$$

Based upon the instantaneous values of the plasticity variables $C_{ij}$ and $D_i$, the strength of each lateral or feed-forward synaptic weight, $\Delta g_{ij}^\mathrm{EE}(t)$, is then modified according to Eq. 6 and governed by the time course variable $\tau _{\Delta g}$.

6 $$\begin{aligned} \tau _{\Delta g}\dfrac{d\Delta g_{ij}^\mathrm{EE}(t)}{\hbox {d}t}&= \underbrace{\left( 1 - \Delta g_{ij}^\mathrm{EE}(t)\right) C_{ij}(t)\sum _p\delta \left( t-t_{i}^p\right) }_\mathrm{LTP}\nonumber \\&- \underbrace{\Delta g_{ij}^\mathrm{EE}(t) D_i(t)\sum _q\delta \left( t-t_{j}^q\right) }_\mathrm{LTD} \end{aligned}$$

Note that the *post*synaptic spike train (indexed by $p$) is now associated with the *pre*synaptic state variable ($C_{ij}$) and vice-versa. If $C_{ij}$ is high (due to recent presynaptic spikes) at the time of a postsynaptic spike, then the synaptic weight is increased (LTP). Conversely, if $D_i$ is high (from recent postsynaptic spikes) at the time of a presynaptic spike, then the weight is decreased (LTD). Since $\Delta g_{ij}^\mathrm{EE}$ is bounded within the interval $[0, 1]$, the maximum possible change in conductance in Eq. 3 is given by $\lambda$.

Throughout the simulations presented, the default parameter values shown in Table 4 were used for the STDP model (Perrinet et al. 2001), except when they were systematically varied (as indicated) to assess their effect upon network performance.

This system of differential equations describing the dynamics of the cell bodies, synaptic conductances and synaptic plasticity is discretised with a Forward Euler numerical scheme, written in the C programming language and simulated with a numerical time step $\Delta t$ of 0.02 ms.

### Stimuli and training

**Fig. 2:**
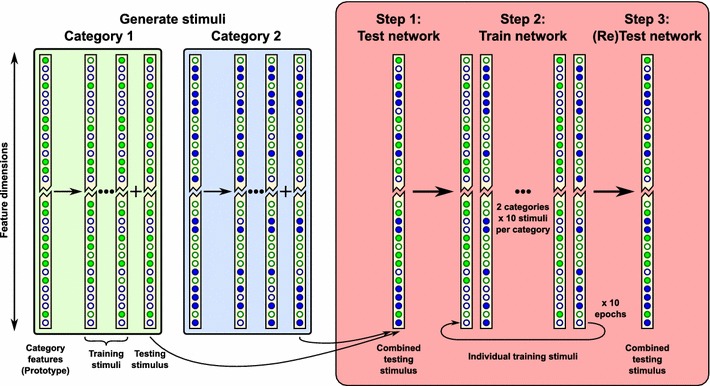
Illustration of the static stimuli used in the one-layer network. The input layer contains 512 neurons (truncated for illustrative purposes) and is divided randomly into two sets: Category 1 (*green*) and Category 2 (*blue*). The 256 input neurons assigned to each category represent the set of visual features defining that category—their respective ‘prototypes’. From each category, ten training stimuli and one testing stimulus are constructed by randomly selecting 128 prototype features. During training, the ten training stimuli from each category are presented in a random order, for a total of ten epochs. The test stimuli from each of the two categories are combined to form a compound test pattern, which represents a scene containing multiple stimuli from different categories. This protocol assesses how training the network on the individual training stimuli from the two categories subsequently enables the network to perform temporal segmentation of the two novel stimuli presented together in the compound test pattern

Stimuli were represented by injecting tonic current into sets of input layer neurons drawn randomly from separate pools, where each pool corresponded to a different category of stimulus, as illustrated in Fig. 2. In the initial one-layer simulations, the input layer consisted of 512 neurons which were divided evenly between the two categories of stimuli into two pools of 256 neurons each, with each example consisting of 128 neurons drawn randomly from one of these pools. Eleven such stimuli were drawn from each of the two stimulus category pools, of which ten from each category were presented to the network sequentially during training (for 500 ms each) for a total of ten epochs. The two reserved stimuli (one from each stimulus category) were combined to present to the network during testing (for 1,000 ms) in order to assess the network’s ability to segment two novel members of the categories before and after learning from the training examples.

**Fig. 3:**
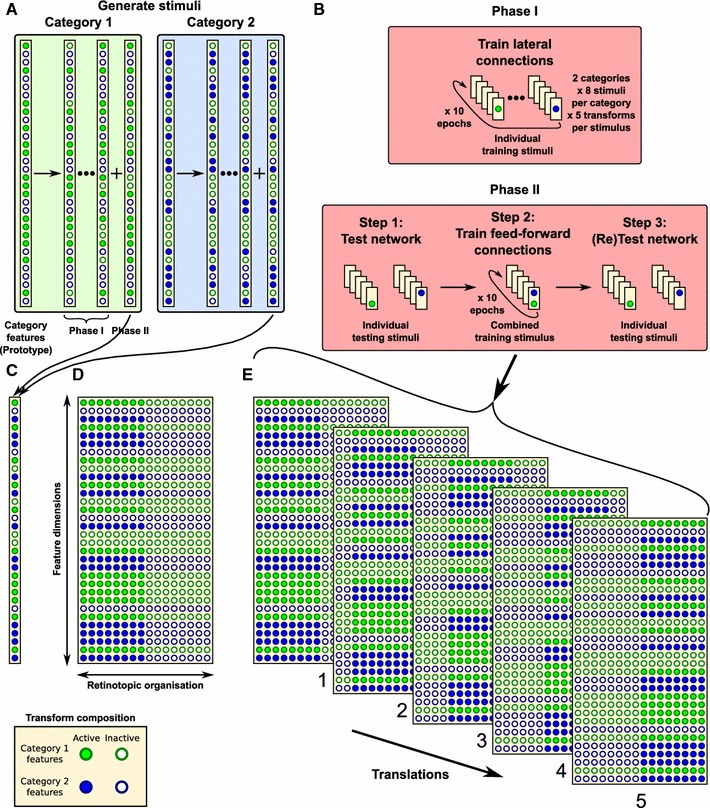
Illustration of the translating stimuli used in the two-layer network. The input layer is 2-dimensional, being comprised of 32 rows $\times$ 16 columns, permitting the stimulus to be translated across the columns. Each stimulus is constructed to have a unique distributed pattern across the 32 rows, which is then extended across 8 columns (i.e. half of the input layer). A ‘prototype’ is first constructed for each of the two categories by randomly selecting 16 out of 32 neurons comprising one column (**a**). For each category, eight stimuli are constructed for Phase I of the simulation, and one extra novel stimulus for Phase II, by randomly drawing 12 from the 16 features of their category prototypes. The first simulation phase trains the lateral connections within the input layer, while the second phase then trains (and tests) the feed-forward connections from the input layer to the output layer (**b**). In Phase I, the lateral connections of the network are trained by presenting eight stimuli from each category (in random order) translating across the columns of the input layer for ten epochs. This training allows the lateral connections to segment novel stimuli from different categories, when subsequently presented together within a scene. Phase II takes place in three steps as follows. In Step 1, the network is tested by individually presenting the remaining (novel) stimulus from each category translating across the input layer. In Step 2, the novel stimuli from the two categories are then combined to form a compound training pattern (**c**), which is expanded over 8 columns as described (**d**). The compound pattern is translated in five overlapping positions across the columns for ten epochs to train the feed-forward connections (**e**). In Step 3, the same two category stimuli are once more presented individually to test whether training the feed-forward connections in Step 2 has enabled the output neurons to develop separate translation-invariant representations of the two stimuli

For the two-layer network, the simulations were organised into two phases, as illustrated in Fig. 3. During Phase I, eight stimuli were presented from each of the two stimulus categories individually translating across the input layer (for five translations each) to train the lateral connections. During Phase II, the lateral ($ElE$) weights were fixed and the feed-forward ($EfE$) weights were trained with two more novel stimuli (one from each stimulus category), presented together as they translated across the input layer for ten epochs. Unlike in the one-layer simulations, the testing stimuli were presented individually so that output cells’ responses could be recorded to each transform of each stimulus independently, in order to analyse their tolerance to translations.

A similar procedure was used to generate training and testing stimuli as for the one-layer simulations, with the main modification being to extend the input layer into a two-dimensional structure of $32 \times 16$ neurons. The 32 rows of the input layer were divided into two equal groups, demarcated in Fig. 3 with green or blue borders. Each stimulus consisted of neurons in twelve rows chosen at random from their particular pool of (16) category neurons (except when category overlap was explicitly investigated as described later). These rows of ‘features’ were extended to be eight neurons wide (totalling 96 active neurons) consisting of columns 1–8 for Transform 1 and shown with solid coloured faces. Each of the subsequent four translations was produced by a shift of two neurons across the rows, ensuring a consistent overlap of 75 % (six columns) between successive translations. This enabled the stimuli to translate in a direction orthogonal to that which defined the stimulus identity, so that the overlap between translations could be controlled independently.

**Fig. 4:**
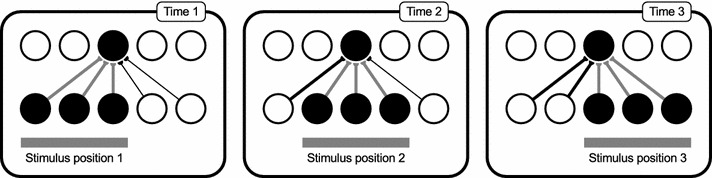
Forming transformation-invariance with the CT learning mechanism. In an untrained network, the initial transform (Time 1) will stimulate the corresponding input neurons, which randomly activate one or more postsynaptic neurons (due to the random synaptic weight initialisation). These particular synaptic connections between the active input and output neurons are then strengthened through Hebbian associative learning (shown in *grey*). If the second transform (Time 2) is similar enough to the first, the shared, previously potentiated afferent connections will encourage the same postsynaptic neurons to fire. This causes the input neurons of the second transform to have their synapses potentiated onto the same set of output neurons through associative learning. This process may continue (Time 3) until there is very little or no resemblance between the current and the initial transforms, provided the network has been exposed to sufficient intermediate transforms. In addition to changes in retinal location, the same principles will apply to build invariance to other types of transformation such as view or scale, provided there is sufficient overlap between the features present in the set of transforms

Ensuring a degree of overlap between the transforms (translations) of a stimulus enables the Continuous Transformation (CT) learning mechanism to associate them together onto the same postsynaptic neurons in the output layer (Stringer et al. 2006; Evans and Stringer 2012). Similar transforms (e.g. overlapping translations) stimulate some of the same presynaptic neurons, thereby likely activating the same set of postsynaptic neurons. Through a local Hebbian process of synaptic modification, the new inputs of these similar transforms then become associated onto the same output neurons, thus leading to the development of transformation-invariant visual representations in a biologically plausible way (Fig. 4).

In contrast to earlier work (Miconi and VanRullen 2010; Evans and Stringer 2013), the input neurons in both the one- and two-layer simulations had no hard-wired topological connectivity with which the input layer could organise the stimuli into perceptual cycles. Instead, the excitatory lateral connections had to self-organise through the experience of commonly coactive features in order to reliably segment novel examples of previously seen categories. Importantly, this allows the investigation of the formation of visual categories according to the statistical associations of visual features, through which individual objects may be recognised in natural scenes.

### Network performance measures

The network performance is primarily assessed using two measures derived from information theory (Bishop 1997; MacKay 2003), which reflect how well cells respond invariantly to a particular stimulus over several transforms but differently to other stimuli (Rolls et al. 1997; Rolls and Milward 2000; Elliffe et al. 2002). In so doing, these analyses measure the extent to which a cell possesses both *specificity* to the identity of a particular object (ideally by responding to one stimulus only) and *generality* to natural variations in its appearance (ideally by responding to all transforms of that stimulus)—the computational crux of visual object recognition (DiCarlo et al. 2012).

While spiking dynamics are critical for how the network organises the stimulus representations, analysis of macaque visual cortical neuron responses has revealed that the majority of information about stimulus identity is contained within the *firing rates* rather than the detailed timing of spikes (Tovée et al. 1994). Accordingly, the network self-organises through spiking dynamics, but the information content (with respect to stimulus identity) is assessed through the output cell’s firing rates.

To measure the information conveyed by the responses of the output neurons, each transform of each stimulus was presented to the input layer of the network individually during a testing phase. Each neuron was allowed to settle after presentation of each transform, such that the activity due to one transform did not affect the responses to later transforms. The spikes of each output neuron were binned individually for each transform of each stimulus and the corresponding firing rate for each cell was calculated. Each cell’s responses were then used to construct conditional $P(r\vert s)$ and unconditional $P(r)$ firing rate distributions. From these distributions, the stimulus-specific single-cell information, $I(s,R)$, was calculated according to Eq. 7. This measure quantifies the information conveyed by a particular cell through its complete set of responses to every transform of every stimulus, $R$, about a specific stimulus, $s$.

7 $$\begin{aligned} I(s,R) = \sum _{r\in R}P(r\vert s)\log _2\frac{P(r\vert s)}{P(r)} \end{aligned}$$

Good performance for a cell is indicated by a high (or maximal) information score, which would entail stimulus specificity, with generality across most (or all) transforms of that stimulus. In terms of the original firing rates, this would mean a large response to one stimulus regardless of its position (transform) and small responses to transforms of other stimuli. Such a cell may transmit relatively little information about other, non-preferred stimuli (for example, by responding indiscriminately to a number of other stimuli or unevenly to their transforms) but will still be very useful if it conveys maximum information for one particular stimulus. We therefore compute the maximum amount of information a neuron conveys about *any* of the stimuli rather than the average amount it conveys about the whole set of stimuli, $S$ (which would be the mutual information).

If all the output cells learnt to respond to the same stimulus, then there would be no discriminability and the information about the set of stimuli ($S$) would be poor. To test this, the multiple cell information measure is used which calculates the information about the set of stimuli from a population of up to $C_{\max } = 5\cdot \vert S \vert$ output neurons. This population consisted of the subset of up to five cells which had, according to the single-cell measure, the most information about each of the two stimuli.

Ideally, we would calculate the mutual information—the average amount of information about which stimulus was shown from the responses of all cells after a single presentation of a stimulus, averaged across all stimuli. However, the high dimensionality of the neural response space and the limited sampling of these distributions are prohibitive to such an approach. Instead, a decoding procedure is used to estimate the stimulus $s^{\prime }$ that gave rise to the particular firing rate response vector on each trial, as detailed below. Knowing (*a priori*) which stimuli have been presented, a probability table (confusion matrix) may be constructed (in the much lower dimensional space) between the real stimuli *s* and the decoded stimuli $s^{\prime }$, from which the mutual information is then calculated (Eq. 8).

8 $$\begin{aligned} I(s,s') = \sum _{s,s'\in S}P(s,s')\log _2\frac{P(s,s')}{P(s)P(s')} \end{aligned}$$

In this work, a Bayesian decoding procedure is used to infer the presented stimulus from the neural responses. For each cell in the ensemble vector, its firing rate response to each unknown transform is separately fitted to a Gaussian distribution of firing rates to each stimulus. Each stimulus-conditional distribution is parameterised by the mean and standard deviation of the cell’s sets of responses to transforms of each particular stimulus. Importantly, the unknown response is excluded from these parameterisations; hence, a jack-knife cross-validation procedure is incorporated in the decoding process. This unknown response is then decoded by comparing it to each stimulus-conditional firing rate distribution to calculate from which it was most likely to have come and so yield an estimate of $P(r_c\vert s')$. Taking the product of these probabilities over all cells in the response vector ($\mathbf {r}$) with $P(s')$ and then normalising the resultant joint probability distribution gives an estimate of $P(s'\vert \mathbf {r})$ (Földiák 1993).

The calculated mutual information values were then corrected to compensate for the upward bias due to finite sampling (Treves and Panzeri 1995). As in previous work, only the first term of an analytically derived series was used, since this has been shown to be a good approximation (Panzeri and Treves 1996; Sugase et al. 1999). To smooth out the effects of random sampling for the neural ensemble, the information values were averaged over $N_i = 100\cdot (C_{\max }-c+1)$ iterations, decreasing linearly (in this case from 1,000 to 100) as the ensemble size, $c$, increases. The smoothed values were then clipped at the theoretical information limits to remove any artefacts caused by the approximate correction terms, before factoring them into the probability tables, $P(s,s')$. From these decoding, cross-validation and correction procedures, more reliable estimates of the true probabilities are obtained for calculating the multiple cell information measure (Rolls et al. 1997).

This multiple cell information measure should increase up to the theoretical maximum $\log _2 N_\mathrm{S}$ bits (where $N_\mathrm{S}$ is the number of stimuli), as a larger population of cells is used, only if those cells have become tuned to different stimuli. A high information score from the multiple cell measure therefore indicates that all stimuli are represented in the ensemble of output cells, meaning that the network has good discriminability.

To assess the network performance across a range of parameter values, an ‘information score’, $\iota _\kappa$ was calculated from the single-cell information described in Eq. 7. For each stimulus, $s$, the number of cells which conveyed at least 95 % ($\kappa = 0.95$) of the theoretical maximum information (in this case 0.95 bits) according to the single-cell measure was counted. The minimum number of such cells for any stimulus in the set was then found and normalised to a proportion of the total number of output cells. This ‘information score’ therefore expresses the information conveyed by the network about all transforms of the least well represented stimulus (see Eq. 9).

9 $$\begin{aligned} \iota _\kappa = \frac{\min _s|\left\{ I_{c,s}\ge \kappa \cdot \log _2N_\mathrm{S}\right\} _c|}{C} \end{aligned}$$

Here, $I_{c,s}$ is the amount of information conveyed by a particular output cell, $c$, about a particular stimulus, $s$ according to the single-cell information measure, $N_\mathrm{S}$ is the number of stimuli and $C$ is the total size of (number of cells in) the output layer. Although this measure is derived from the single-cell information measure, taking the minimum proportion of cells across all stimuli means that nonzero values of $\iota _{\kappa }$ indicate that all stimuli are represented, fulfilling the role of the multiple cell information analysis.

## Results

In the first section of results (Sect. 3.1), we explore the formation of perceptual cycles in a single layer of laterally connected excitatory and inhibitory neurons. The lateral excitatory to excitatory connections ($ElE$) are first trained through exposure to examples of each stimulus category presented individually. The network is then tested with a *novel* example from each category presented simultaneously. Its behaviour during this presentation is analysed to determine whether it was able to perceptually organise the novel stimuli, by representing each alternately through time in anti-phase oscillations.

In the second section of results (Sect. 3.2), this work is extended to the case of translating stimuli which are presented individually and shifted (in lockstep) across the input layer during training of the lateral connections. A second output layer of excitatory and inhibitory neurons was added to the network with plastic feed-forward synapses from the excitatory neurons in the input layer to the excitatory neurons in the output layer ($EfE$). Training of the lateral connections ($ElE$) proceeded as in the single-layer simulations (except with moving stimuli). The recurrent weights were then fixed, and the feed-forward weights were trained on the novel examples translating *together* across the input layer. The network was then tested by presenting each novel stimulus *individually* translating across the input layer. The network’s output neurons were then analysed for their ability to recognise each novel stimulus across all of its transforms using the information analysis techniques described (Eqs. 7–9).

### Learning to segment novel stimuli

**Fig. 5:**
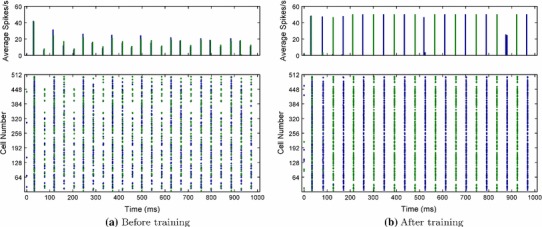
Excitatory neuron activity before and after training the excitatory lateral connections. The *colours* demarcate the two populations of principal cells which constitute the novel examples from each of the two stimulus categories. Both histograms of the binned average spike rates (*top*) and spike rasters (*bottom*) are shown for each population of neurons. Prior to learning, both populations are rapidly synchronised through the action of the fully connected inhibitory interneurons (global inhibition). After learning, it can be seen that each population of neurons is internally synchronised and desynchronised with respect to the other population

The first results presented are a demonstration of how plasticity in lateral excitatory connections enables temporal segmentation by anti-phase oscillation of novel examples belonging to previously seen categories. This is evident in the raster plots of Fig. 5. Prior to training, when presented with two novel testing examples simultaneously (one from each category), the excitatory neurons from both categories exhibited global synchronisation driven by the effects of the inhibitory interneurons (Fig. 5a). After training the plastic $ElE$ connections on ten examples from each of the two categories, the presentation of the same combination of novel examples then resulted in the generation of perceptual cycles. That is, the two populations of excitatory neurons representing each novel example synchronised their firing with respect to other neurons in their group but fired in opposite phase with respect to neurons in the other group (Fig. 5b). This can also be observed in the histograms where the two colours denote the binned average spike rates for the two groups of neurons (Fig. 5, top row).

**Fig. 6:**
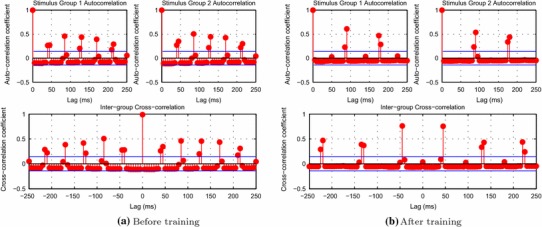
Autocorrelations for each neural group corresponding to each example (*top row*) and Cross-correlation between groups (*bottom row*) before and after training. (The *blue lines* are the approximate upper and lower 95 % confidence bounds, assuming that time series are completely uncorrelated.) Only positive lags are shown for the autocorrelation functions since they are symmetric about 0. Before training, neurons from both groups (stimulus categories) fire synchronously (as indicated by the strong positive cross-correlation at zero-lag), with a regular period of approximately 40–45 ms. After training, however, the period of firing for each group has doubled to approximately 85 ms with each neural group firing in opposite phase to one another as indicated by the very small zero-lag cross-correlation

The self-organisation through synaptic modification can be further seen in the auto- and cross-correlations shown in Fig. 6. Before training, the neurons of both groups fire with a regular period of approximately 40–45 ms, as evidenced by the regular significant peaks in the autocorrelations of Fig. 6a. The cross-correlation before training exhibits the same periodicity as the autocorrelations and also has a large positive correlation at zero-lag, indicating that both groups of neurons are firing in phase with one another. After training, however, the period of the autocorrelations for each stimulus group can be seen to have doubled to approximately 85 ms, demonstrating that each group still has periodic (oscillatory) firing but now each fires at half the frequency of the untrained case (Fig. 6b). Additionally, the cross-correlation also now shows double the period between significant correlation peaks and no longer has a significant correlation at zero-lag but at $\pm$40–45 ms lag. This indicates that neurons of each stimulus group are no longer firing synchronously with the other group, but are firing on alternative cycles of approximately 22 Hz (while synchronised with members of their own group).

**Fig. 7:**
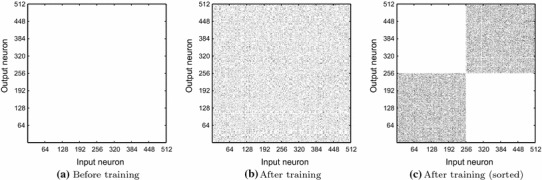
Excitatory lateral synaptic weights ($\Delta g_{ij}^\mathrm{ElE}$). Before training, the lateral weights (synaptic efficacies) have no structure as they are uniformly initialised to 0. After training, some of the weights have increased due to the synchronised firing of input representations. After sorting the weights according to stimulus category membership, the structure of the weight matrix is made clear. The stimulus features of Category 1 have formed strong connections with the other features of their category exclusively (*bottom left quadrant*) while the features of Category 2 have made strong connections with the other features of their category

From examining the weight matrices (shown in Fig. 7), the reason for this organisation of the input representations is made clear. Before training (Fig. 7a), there was no structure in the excitatory–excitatory lateral connections, as all synaptic efficacies ($\Delta g_{ij}^\mathrm{ElE}$) were initialised to 0. After training, some of the weights can be seen to have increased (Fig. 7b) as expected from the synchronous firing of the particular principal cells representing features of their stimulus category. After reordering the rows and columns (representing the post- and presynaptic excitatory cells) of the post-training weight matrix according to category membership (Fig. 7c), the structure developed in the lateral connections is made apparent. Since the features drawn from a particular category were always experienced together during exposure to the ten examples of that category during training, strong connections formed between these features. This is evident for Group 1 in the sorted lower left quadrant of the post-training weight matrix and for Group 2 in the upper right quadrant (Fig. 7c).

Since these strengthened lateral connections were effectively shaped through the course of training according to the statistics of the co-occurrence of visual features, the connections were strengthened between neurons representing features from the same stimulus category. Then when multiple novel stimuli from each of the two stimulus categories were presented together after training the lateral connections (even without spatial separation), neurons representing the features of one category acted in a mutually supportive way to synchronise the firing of neurons representing other visual features present from the same category. The firing of each corresponding neural group competed to suppress the other through the inhibitory interneurons until only one group of principal cells remained active. Combined with cell firing-rate adaptation acting as a mechanism of self-inhibition, these effects generated perceptual cycles when novel examples with visual features characteristic of their particular categories were presented after training (as demonstrated in Figs. 5 and 6).

#### Strength of lateral connections

The key function of the networks of input neurons investigated here is to synchronise the neurons representing the features of one stimulus category and desynchronise them with respect to the others, allowing them to alternate in an anti-phase relationship dubbed ‘perceptual cycles’ (Miconi and VanRullen 2010). To quantify the network’s ability to self-organise in this way, we applied the same measure as Miconi and VanRullen which calculates correlations in spiking activity between the two (or more) populations representing each category and between sub-populations of the same category for the ‘between-stimuli’ and ‘within-stimuli’ measures, respectively.

During the testing phase (with synaptic plasticity turned off), the network was presented with the two novel stimuli simultaneously (one from each category) for 1,000 ms. The spikes from each corresponding group of neurons were put into 10 ms bins, and those bins containing less than ten spikes were excluded to prevent quiescent periods from degrading the performance measure (Miconi and VanRullen 2010). The correlation between this frequency series was then calculated with Spearman’s rank method (Miconi, personal communication), yielding the between-stimuli correlation measure. Similarly, each group of neurons was divided in two, and the same procedure was repeated for the two halves to obtain a within-stimulus correlation for each group, that was then averaged across all groups to give the final within-stimuli measure.

For these simulations, the standard one-layer network was used and tested over a range of strengths of the maximum lateral excitatory connections, $\lambda ^\mathrm{ElE}$, from 0.05 to 500 nS (the upper bound which these connections may reach through learning). This range of parameters was repeated for a total of ten random seeds to gauge the effects of statistical variation and the *intergroup*, and *intragroup* synchrony measures were calculated as described. The means across the random seeds were calculated and plotted with error bars representing the standard error of the means.

**Fig. 8:**
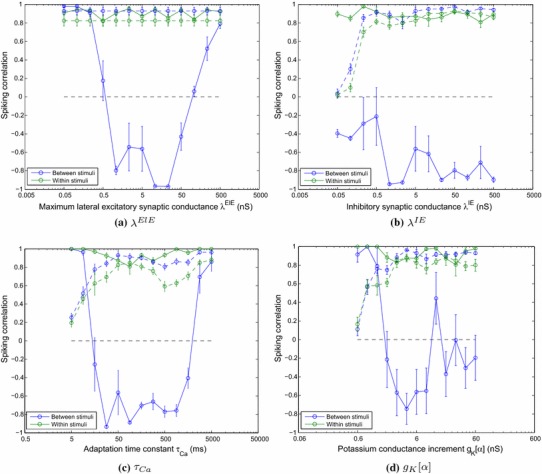
The spiking correlation measure within and between stimuli (with the corresponding pre-training measures plotted with *broken lines*) plotted against **a** maximum lateral excitatory conductance strengths **b** lateral inhibitory conduction strengths **c** adaptation time constants and **d** potassium current increments, each on a logarithmic scale. Each parameter value was simulated ten times with different random seeds with the *data points* representing the mean values across random seeds and the *whiskers* indicating the standard error of the mean. The anti-phase representations were found to be a robust phenomenon over approximately **a** two orders of magnitude with respect to maximum lateral excitatory strength, **b** four orders of magnitude with respect to inhibitory strength, **c** two orders of magnitude with respect to the adaptation time constant and **d** one order of magnitude with respect to the strength of the adaptation mechanism

It can be seen from Fig. 8a that while the synchrony measures within and between the stimuli are very similar before training, the effect of learning in the lateral connections is to make the correlation between stimuli strongly negative (anti-phase), while maintaining a high within-stimuli correlation measure, over a broad range of conductances of approximately two orders of magnitude. When the conductances become too low, the synaptic efficacies are not strong enough to synchronise the neurons belonging to the same stimulus category and so the effect tends to resemble that of no training. When the conductances are too high, the effect on the correlation measure is similar (converging the two measures in global synchrony) but for a different reason. Very strong conductances saturate the firing rates of the neurons, overwhelming the inhibitory interneurons and firing-rate adaptation such that all excitatory neurons in the whole layer are firing near their upper limit and hence are in global synchrony.

In much previous work, inhibitory interneurons have been found to play an important role in synchronising volleys of excitatory cells. Earlier research (not shown here) suggests that modelling inhibitory cells as hyperpolarising makes the system very sensitive to the strength of inhibition, requiring careful retuning for different simulation conditions. Conversely, shunting inhibitory interneurons have been found to produce more consistent effects with respect to network oscillations and excitatory synchrony. We present the effects of variation in the inhibitory conductance strength $\lambda ^\mathrm{IE}\Delta g$ of the shunting interneurons.

It can be seen that before training, the network loses its ability to synchronise the volleys of excitatory action potentials for inhibitory conductances at approximately 10 % or less of the strength of excitatory conductances. Through the training regime, however, the lateral excitatory conductances become sufficient to form the anti-phase input representations (albeit with less consistency than at higher values of inhibitory conduction strength). Figure 8b also illustrates that for very large inhibitory to excitatory conductances, the network still performs well, demonstrating that the strength of these synapses is not important for achieving perceptual cycles in this experimental paradigm (although inspection of the raster plots revealed that the period of oscillations is extended in this case).

#### Adaptation

As found in previous work, a model of self-inhibition, such as firing-rate adaptation, is crucial to generating perceptual cycles (Miconi and VanRullen 2010). The parameters governing the firing-rate adaptation are therefore key to the self-organisation of the network, and thus, parameter variations are explored here in the same way.

The first parameter governing the cell firing-rate adaptation explored here is the calcium decay time constant $\tau _\mathrm{Ca}$. This adaptation time constant determines the time course over which the intracellular calcium dissipates and hence how quickly the neuron recovers from the additional membrane leakage (adaptation current) caused by the calcium-gated potassium channels. With a short time constant, the calcium clears quickly and hence the potassium current acting against perturbations from resting potential quickly returns to nothing.

Figure 8c shows the effect of varying the adaptation time constant $\tau _\mathrm{Ca}$ from 5 to 5,000 ms on the spiking correlation measures. The means of the results of ten randomly initialised simulations are plotted along with the standard error of the mean, indicated by the whiskers at each point. The results demonstrate that the formation of anti-phase input representations is robust over approximately two orders of magnitude of variations in the adaptation time constant (approximately 20–2,000 ms).

Inspection of the spike rasters reveals that with very short time constants, the firing-rate adaptation is too fleeting for the neural groups to self-inhibit, so all neurons fire synchronously. As the time constant becomes too large, the groups do fire in anti-phase but are so strongly adapted (inhibited) by their calcium-mediated potassium currents that the oscillations occur with a very low frequency. As such, there may only be one volley per group in the entire 1,000 ms period (after the initial inter-group synchronised volleys) which leads to very low performance according to the spiking correlation measure.

The second adaptation parameter explored here is the effective increase in potassium conductance due to a single action potential, $g_\mathrm{K}[\alpha ]$, mediated by the intra-cellular calcium concentration. Larger potassium conductances mean that larger potassium currents leak through the cell membrane, making the membrane potential more resistant to larger perturbations from its resting potential (either depolarising or hyperpolarising). In effect, the greater inertia in the membrane potential results in the neuron being more difficult to excite to its firing threshold. Conversely, smaller potassium conductances mean that the self-inhibiting effects of the adaptation (potassium) current are weaker so the firing rate does not decrease as much during constant (super-threshold) excitatory stimulation.

In a similar way to the analysis of the adaptation time constant, the spiking correlation measure was calculated for a range of potassium current increments ($g_\mathrm{K}[\alpha ]$) from 0.6 to 60 nS. Figure 8d shows the effect of varying this parameter on the spiking correlation measure within and between stimulus categories.

As expected, for very low values of $g_\mathrm{K}[\alpha ]$, the effect of the adaptation current is weakened to the extent that the degree of adaptation is negligible, much like the case of very short adaptation time constants. Correspondingly, the raster plots in such conditions show that the groups of neurons from both stimulus categories are firing synchronously (as the dominant mechanism becomes the synchronising force of the inhibitory interneurons). With very large potassium current increments, the firing-rate adaptation becomes very strong, slowing the spike rate for both populations of neurons. Under these conditions, the network is still reasonably capable of forming anti-phase representations, since qualitatively there is still a self-inhibitory mechanism. However, quantitatively, the effects of adaptation become so strong as to reduce the period of oscillations, effectively reducing performance due to the ever fewer number of cycles to measure within the 1,000 ms testing period.

### Learning transformation-invariant representations of novel stimuli

Having explored the conditions under which anti-phase representations may arise through lateral synaptic plasticity in a single-layer model, this section extends this work to a two-layer model. Plastic feed-forward connections are incorporated from the excitatory cells in the input layer to those in the output layer, in order to investigate how these input representations subsequently shape the output representations. The working hypothesis is that the spike-time sensitive learning rule in the feed-forward connections will exploit the time gaps between the volleys of spikes from each stimulus (even as they translate across the input layer), to learn about one stimulus without interference from the other. Thus, although the two translating stimuli are presented to the network simultaneously, without spatial separation and moving in lockstep, prior learning in the lateral connections should allow separate transformation-invariant representations of each stimulus to form in the output layer. Importantly, this is achieved through the richer dynamics of spiking neurons by a mechanism unavailable to rate-coded models.

**Fig. 9:**
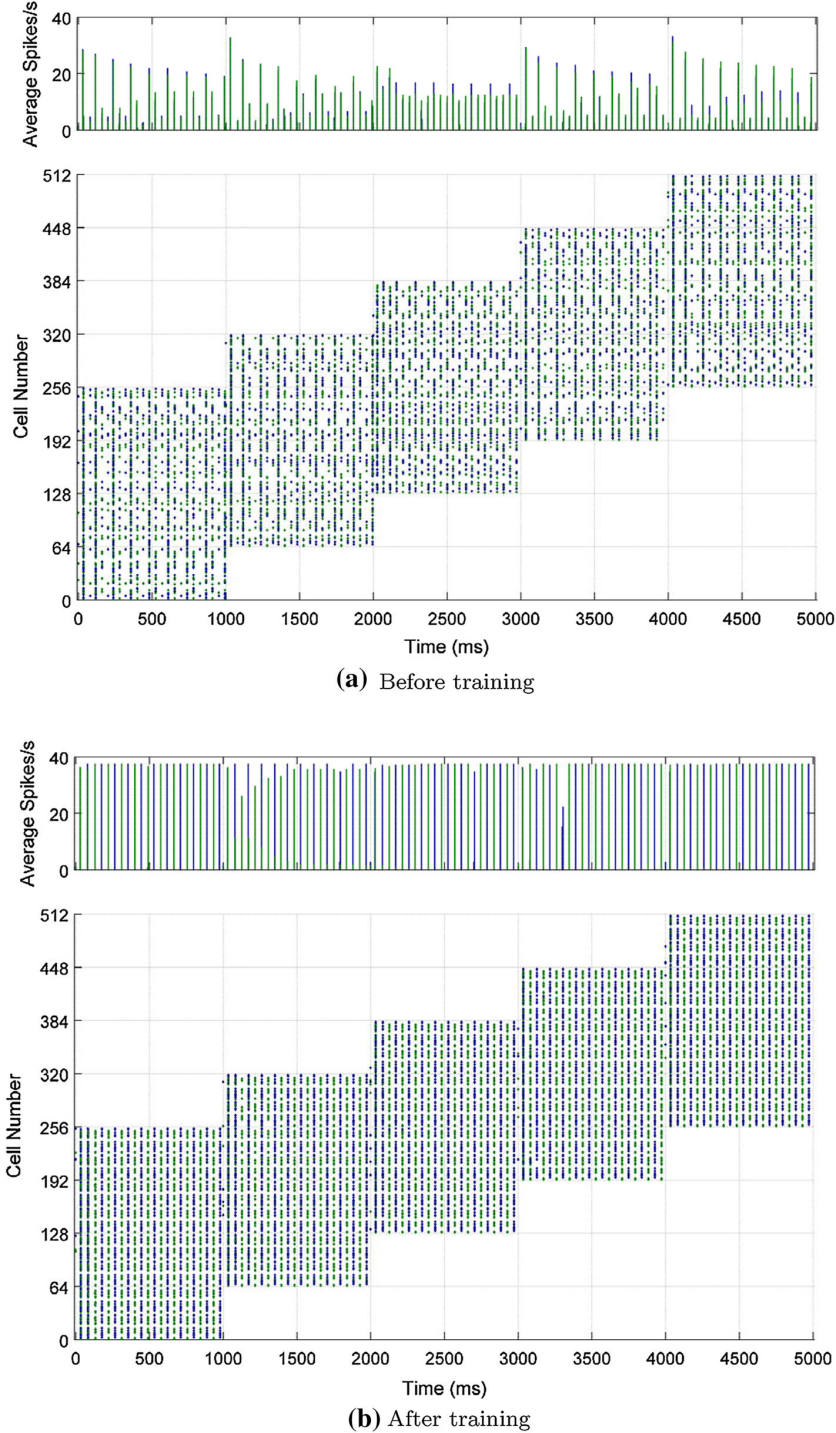
Input layer excitatory neurons before and after training the excitatory lateral connections. A novel pair of stimuli, one from each category, are presented together translating across the input layer. Since the two populations of neurons representing the two stimuli are spatially intermixed and translate together, they are represented by *different colours*. Prior to learning, both neural populations (representing each of the novel examples) are rapidly synchronised through the action of the fully connected inhibitory interneurons (global inhibition), shown by histograms of binned average spike rates (*top*) and spike rasters (*bottom*). After learning, it can be seen that each population of neurons is internally synchronised and desynchronised with respect to the other population

The training regime was adjusted for the multiple layer model so that stimuli translated across the input layer for a total of five transforms. While consecutive transforms are significantly overlapping in order to build translation-invariance through the CT effect, the first and the last transforms of a particular stimulus are completely orthogonal (which may be seen in the rasters of Fig. 9). The training period also consisted of two phases, the first of which trained the input layer lateral excitatory connections exclusively (Phase I) while the second trained only the excitatory feed-forward connections between the layers (Phase II). This two-stage training represents the early learning of categories through exposure to representative example stimuli, followed by more recent learning of specific novel examples in a transformation-invariant manner.

During Phase I, eight training stimuli from each stimulus category were presented individually translating across the input layer for ten epochs while the stimulus categories were encoded in the lateral excitatory connections (illustrated in Fig. 2). During Phase II, the novel stimuli were presented as a pair translating across the input layer for another ten epochs while the excitatory feed-forward connections were trained (illustrated in Fig. 3). Before and after this training phase, the network was presented with each of the novel stimuli individually translating across the input layer in order to test and record the output layer neurons’ responses to each transform separately. The responses were then analysed for stimulus selectivity and transformation-invariance. An example where this was achieved through training is presented below.

For the first simulations, additional testing phases were used to characterise the effects of the preliminary training phase, as shown in Fig. 9. The figure shows the spike rasters of the input layer neurons as a novel pair of stimuli from the two stimulus categories are presented together translating across the input layer before training (Fig. 9a) and after training (Fig. 9b). Similar to the single-layer simulations (Sect. 3.1), the process of training changes the input volleys from disorganised fragments of different stimuli firing synchronously (Fig. 9a) to the anti-phase oscillations of each stimulus represented in turn (Fig. 9b). This dynamic of perceptual cycles is sustained as the stimuli translate together across the input layer, such that they remain temporally separate despite their concerted movement.

With perceptual cycles of the combined novel stimuli established in the inputs, the feed-forward connections between the layers of excitatory cells ($EfE$) are able to exploit this organisation (temporal separation of stimuli) through STDP during the secondary training phase. Even though the network was presented with both novel stimuli simultaneously translating across the input layer, most output neurons have learnt to respond discriminatingly and transformation-invariantly to one stimulus or the other.

**Fig. 10:**
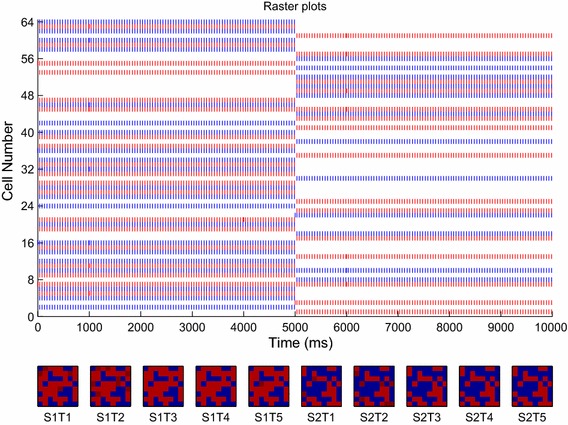
Raster plot of the output layer (*top*) and firing rate plots (*bottom*) for each transform of each stimulus in the output layer after training. Some excitatory cells in the output layer have become responsive to each transform of Stimulus 1, presented over the first 5,000 ms, while other excitatory output cells have become responsive to all transforms of Stimulus 2, presented across the second 5,000 ms. Almost all cells have become transformation-invariant to one particular stimulus, with the exceptions of neurons 7, 10 and 45 which respond indiscriminately to both stimuli

This may be seen in the raster plot of the output layer’s excitatory neurons, as shown in Fig. 10. This figure shows the responses of the excitatory output neurons, when each transform of the first stimulus is presented in turn for 1,000 ms (accounting for the first 5,000 ms) followed by each transform of the second stimulus (accounting for the second 5,000 ms). The majority of cells are observed to fire continuously for either the first period (0–5,000 ms) or the second (5,000–10,000 ms), remaining silent for the other period. It is therefore apparent from Fig. 10 that almost all the cells have become selective for one particular stimulus, and that they respond invariantly across all transforms of their preferred stimulus.

The cell firing rate plots of Fig. 10 depict the same information in an alternative form, whereby the transform presentation periods are discretised as individual plots and the firing rates within those periods are represented by the heat map. It can be seen that the same patches of output cells remain active (shown in red) across all transforms of one stimulus, and are then quiescent (shown in blue) during presentations of the other stimulus. In keeping with the raster plots, the two groups of output cells can be seen to be almost mutually exclusive between the two stimuli. From inspecting the feed-forward synaptic weights (not shown), it was found that the initially uniform distribution had become bimodal after training. As expected from a multiplicative form of STDP (i.e. with soft bounds), a wide Gaussian hump had emerged in the distribution, but with a relatively large peak near the maximum end of the weight scale, corresponding to those synapses which had become strongly selective through learning.

**Fig. 11:**
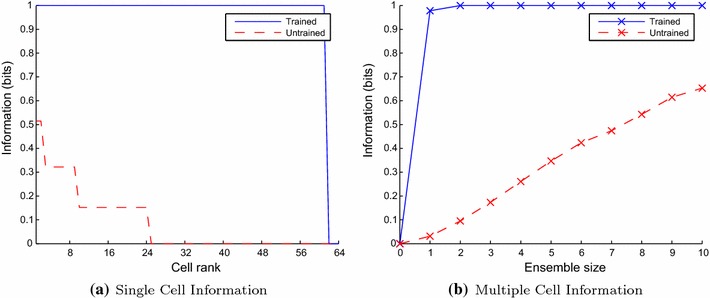
Information plots for the trained and untrained two-layer network. The single-cell information measure (**a**) shows that almost all excitatory neurons in the output layer convey the maximum quantity of information for distinguishing between the two stimuli (1 bit), across all transforms. The multiple cell information measure (**b**) confirms that both stimuli are represented rather than just one, conveying the maximal quantity of information with an ensemble size of just two neurons

After training the feed-forward connections (in Phase II), each stimulus was presented individually to enable the specific responses to each particular transform to be measured. The information conveyed by the firing rates was then quantified according to the single and multiple cell information measures, which is presented in Fig. 11. The single-cell information measure of Fig. 11a shows that almost all neurons convey the theoretical maximum 1 bit of information, meaning that they are perfectly able to distinguish between the two stimuli across all of their transforms. The multiple cell information measure of Fig. 11b shows that of these trained output neurons, some have become responsive to each of the stimuli, such that both stimuli are represented.

#### Overlap between stimulus categories

**Fig. 12:**
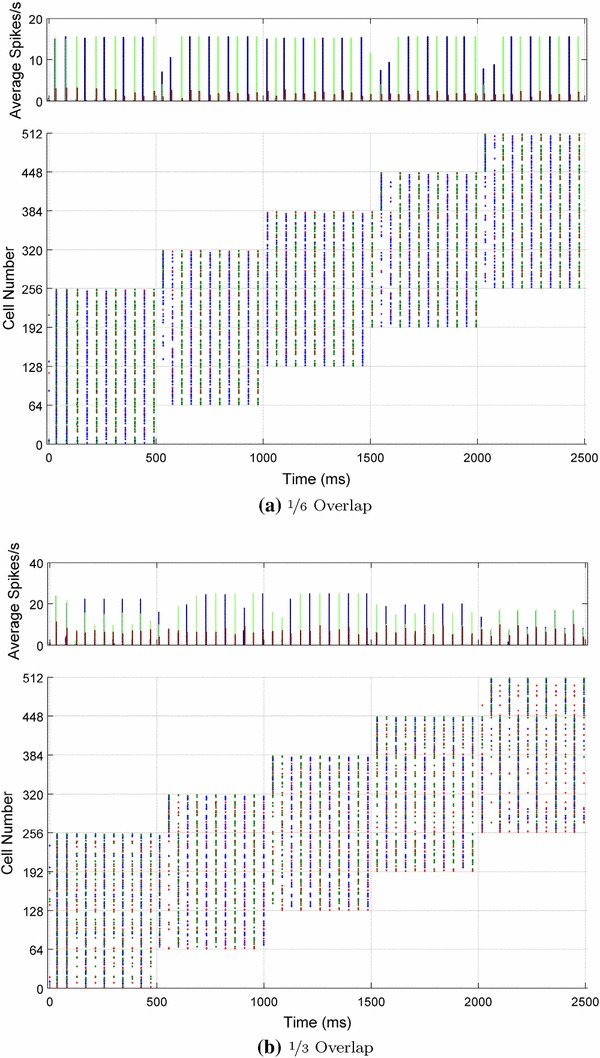
Input layer excitatory neurons after training the lateral excitatory connections for two degrees of stimulus category overlap. A novel pair of stimuli, one from each category, are presented together translating across the input layer during training of the feed-forward connections (500 ms per transform). The category-specific features are demarcated by the colours *blue* and *green*, while shared neurons between the two stimulus categories are shown in red for both the histograms of binned average spike rates (*top*) and the spike rasters (*bottom*). As the overlap increases between the two stimuli, the tendency for them to collapse into one synchronised representations increases, with typically complete loss of segmentation with an overlap of

In this section, the same simulation paradigm was used as in the immediately preceding experiments; however, the two stimulus categories were modified to share some of their features. This was to investigate the expected collapse of the oscillating stimulus representations into one synchronised representation. Figure 12 shows input rasters and post-stimulus time histograms (PSTHs) for the input layer during training of the feed-forward connections for two degrees of overlap.

In particular, for the simulations depicted in Fig. 12a, two rows of input neurons were designated to contain the shared features (present in both stimulus category prototypes) and the remaining 30 rows were divided randomly into two equal groups of 15 category-specific features. The two shared features (rows of neurons) were activated for each training example, along with ten other features chosen randomly from that particular category’s pool of (15) non-shared features. As before, each individual stimulus consisted of twelve rows of neurons extend across eight columns of the input layer, but now with two rows of these rows active in all other stimuli. This meant that the proportion of neurons shared between any pair of example stimuli from the two different categories was $16/96 = 16.7$ % and these neurons are shown in red. While there are occasionally volleys of spikes which are synchronised between groups, particularly at the beginning of each transform, the segmentation of the stimuli is largely robust to this degree of overlap.

The simulations were repeated with an increasing number of neurons shared between the categories. Another illustration is shown in Fig. 12b for the case where four features (rows of input neurons) were shared between the categories, and each example comprised of these with an additional eight features drawn randomly from the 14 features unique to its category. The resulting proportion of shared neurons was therefore increased to $32/96 = 33.3$ %. It can be seen under this condition that more volleys contain neurons representing (unique) features of both categories firing in synchrony and that there is some fragmentation of the representations (for example in the last two transforms). However, information analysis (not shown) revealed that some output neurons were still able to form translation-invariant representations for one novel stimulus or the other for this degree of overlap.

As the overlap increases between the stimulus categories to 50 % and above (not shown), they naturally tend to collapse into one representation. This is achieved primarily through the effects of the reinforcement between the shared features and the unique features of each category in the lateral connections, which tend to synchronise stimuli from different categories rather than segment them. This process is then further reinforced by strengthening all synchronously firing inputs (from each category) onto the same output neurons, forming combined output representations. While these output representations are translation-invariant, they are not able to discriminate between stimuli from the two categories resulting in low multiple cell information measures and information scores. This is a fairly natural expectation, however, as it is essentially the same process which bound examples of the same category together (and previously allowed them to be segmented from examples of a sufficiently different category).

#### Excitatory feed-forward synaptic strength

**Fig. 13:**
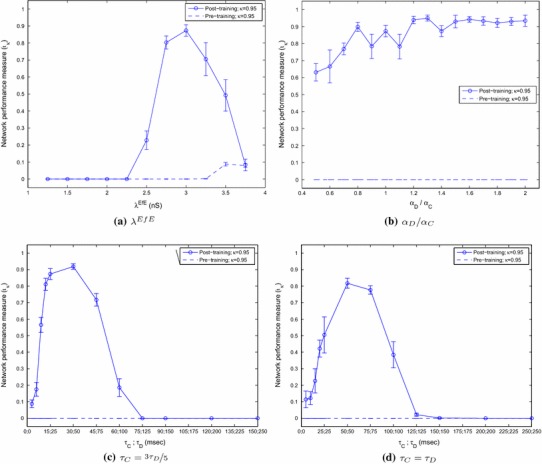
Mean performance ($\iota _\kappa$) of the two-layer network across ten random seeds before and after training plotted against variation in four parameters. The independent parameters explored are **a** the maximum strength of the excitatory feed-forward synapses ($\lambda ^\mathrm{EfE}$); **b** the STDP plasticity $\alpha$-constants ratio (); **c** asymmetric STDP plasticity time constants (with fixed ratio ) and **d** symmetric STDP plasticity time constants ($\tau _\mathrm{C} = \tau _\mathrm{D}$). The network performance measure is evaluated as the lowest percentage of output cells with at least 95 % of maximum information to either stimulus and the *whiskers* denote the measure’s standard error of the mean across the random seeds. In summary, **a** the optimal network performance can be seen to be achieved at $\lambda ^\mathrm{EfE} = 3$ nS; good network performance can be seen to be achieved for **b**  ratios of approximately 0.8 and above; **c** $\{\tau _\mathrm{C} ; \tau _\mathrm{D}\}$ values around $\{12 ; 20\}$ to $\{45 ; 75\}$ ms and **d** symmetric plasticity time constants ($\tau _\mathrm{C} = \tau _\mathrm{D}$) of 50 to 75 ms

While the input representations had self-organised appropriately, the strength of the feed-forward excitatory synapses required some tuning to ensure that there was enough activity to stimulate the network’s output layer, but not so much as to saturate the postsynaptic neurons’ firing rates. The feed-forward synaptic conductance strengths were initialised randomly, uniformly distributed in the range $[0, \lambda ^\mathrm{EfE}]$ and updated (within the same range) according to the same spike-timing-dependent learning rule used for modifying the lateral excitatory connections. To explore the effects of varying the feed-forward synaptic conductance and find an optimal value for $\lambda ^\mathrm{EfE}$, a parameter exploration was conducted over the range 1.25–3.75 nS. Network performance was evaluated as the lowest proportion of output cells with at least 95 % of the theoretical maximum quantity of information ($\log _2N_\mathrm{S}$) to either stimulus, denoted $\iota _{\kappa }$ (see Eq. 9) and is shown across the range of maximum synaptic efficacies in Fig. 13a.

From the mean of ten random seeds, a maximum excitatory feed-forward synaptic strength of 3 nS was found to be optimal for training the output layer across all five transforms. The results of one realisation (particular random seed) of this parameter are presented previously in Figs. 9, 10 and 11. For maximum feed-forward conductance strength values of 2.25 nS or less, the input layer was unable to stimulate output neurons adequately (or at all), such that there were no spike responses to record and hence no information in the quiescence. For large values, typically too much activity was propagated, making it more difficult to distinguish the responses of the output neurons to each stimulus as the firing rates approached saturation point.

#### LTP/LTD plasticity balance

To investigate the sensitivity of the network to the details of the STDP learning rule, the ratio of $\alpha _\mathrm{D}$ to $\alpha _\mathrm{C}$ was systematically varied. This effectively manipulated the relative strengths of the LTP and LTD plasticity components as indicated in Eqs. 4–6. As before, ten random seeds were run for each $\alpha$-ratio in the range $[0.5, 2]$. It can be seen from Fig. 13b that the model is fairly robust to variations in this ratio, particularly for values above . This implies that the network performs best when the LTD component dominates the LTP, potentially helping to prevent spurious reinforcement at synapses due to stochastic fluctuations in spike arrival times.

#### Time constants of synaptic plasticity

Perceptual cycles have been shown to be a robust mechanism for separating simultaneously presented stimuli, even though the ensembles of neurons representing each stimulus receive the same (tonic current) stimulation over the same time course. If the oscillations formed are sufficiently separated in time, a spike-time sensitive learning rule in the connections between layers will enable selective learning about each population of input neurons. As demonstrated, this dynamic will allow output neurons to develop transformation-invariant representations of one stimulus without interference from others.

This mechanism may operate provided that the form of STDP is temporally sensitive enough, such that only the input volley immediately preceding an output neuron’s spike causes potentiation and not prior volleys from the representations of other stimuli. It is expected that if the form of STDP used to modify these inter-layer $EfE$ synapses were made less temporally specific, then the oscillations may occur on a time scale too short for postsynaptic excitatory neurons to learn about each stimulus in isolation. That is, if the time window for potentiation were to encompass consecutive input oscillations representing different stimuli, they would be indiscriminately associated onto the same output neurons.

Conversely, if the time constants of synaptic plasticity are too small (and so the time windows for the STDP are too short), then the spread of spikes within a volley may become too large for the earliest firing neurons to become potentiated onto the same output neuron as those firing later which ultimately cause the postsynaptic neuron to fire. This may mean that the full set of features representing a particular stimulus are not associated together onto the same output neurons, making the network less tolerant to noise and uninformative variations in its inputs.

To test these hypotheses regarding the time constants of the STDP model used ($\tau _\mathrm{C}$ and $\tau _\mathrm{D}$), these parameters were systematically varied while maintaining a constant ratio of . Figure 13c plots the results of simulations varying the STDP time constants from $\{3; 5\}$ to $\{150; 250\}$ ms for ten different random seeds showing the mean network performance (and standard error of the mean) across those random seeds. As expected, the network’s performance is quite sensitive to the STDP time constants, achieving good performance in the region of $\{12; 20\}$ to $\{45; 75\}$ ms, but dropping sharply beyond this range.

The same simulations were rerun but with symmetric time constants ($\tau _\mathrm{C} = \tau _\mathrm{D}$), with the network performance plotted in Fig. 13d. Compared with asymmetric STDP time constants, this parameter change resulted in slightly lower performance overall and also slightly longer time constants of 50 ms to achieve optimal performance over the same ten random seeds. However, in each case, performance dropped off sharply, becoming essentially indistinguishable from an untrained network for STDP time constants around 125 ms and above.

As hypothesised, these results confirm the need for temporal specificity in the STDP learning rule, in order to form separate stimulus representations in the output layer.

## Discussion

In the presented work, it has been demonstrated how introducing plasticity into excitatory lateral connections enables them to encode information about the category to which stimuli belong. This is achieved through associating together co-occurring features within stimuli which typify a particular category. Once modified through exposure to several category members, these lateral connections were then shown to be able to segment a visual scene composed of two *novel* examples (one from each category) by synchronising the features within a particular stimulus and desynchronising each stimulus representation with respect to the other—a dynamic known as ‘perceptual cycles’ (Miconi and VanRullen 2010). In keeping with previous work, a mechanism of delayed self-inhibition was found to be necessary in organising the input representations into perceptual cycles. In this particular work, cell firing-rate adaptation was used for this purpose, which is found to be a common property of cortical neurons.

By augmenting the network with a second output layer of excitatory neurons linked by feed-forward, plastic synaptic connections, the second section of work showed how this encoded category information could facilitate the process of transformation-invariant object recognition. The perceptual cycles generated in the input layer (by the previously learned category training) were exploited through a spike-time sensitive learning rule, such that the output layer learned separate transformation-invariant representations of each novel example. The formation of perceptual cycles (anti-phase oscillations of the input representations) and subsequent network performance in learning independent and invariant stimulus representations were found to be robust to a wide range of several parameters.

In our earlier work (Evans and Stringer 2013), the perceptual cycles between different stimuli were achieved through spatial separation and a ‘Mexican hat’ profile of fixed lateral excitatory connection strengths. In contrast, the same organisation of the input representations was achieved in this paper through encoding prior experience of stimulus categories in the initially weak lateral excitatory connections. This meant that the stimuli did not need to be physically separated, exhibit independent motion or be statistically decoupled by presentation with many other stimuli in order to segment the visual scene and subsequently produce stimulus-specific transformation-invariant output cells.

By replacing the fixed lateral ‘Mexican Hat’ connections with the plastic ones incorporated in this work, the model gains the additional abilities to:

(i) Learn about categories of objects (by associating together the features common to their examples) and encode this information in the lateral connections between the excitatory neurons.
(ii) Utilise prior category knowledge encoded in the lateral connections to temporally segment novel examples of previously experienced categories when presented together.
(iii) Exploit the temporal segmentation in the input layer with STDP in the feed-forward excitatory connections to simultaneously learn independent transformation-invariant representations of novel translating stimuli.

An interesting extension to this work would be to investigate the time course of the different types of information. Since the plasticity in the lateral connections leads to more precise associations between input neurons based upon the statistical features of the stimuli, this may provide an explanation for the time course of information observed in inferotemporal cortex. The features most reliably associated with a particular stimulus category will most likely have the strongest mutual connectivity. Whenever some of them are activated by a particular stimulus, the other most typical features will receive the most lateral excitation and are therefore most likely to fire first, followed later by the more discriminatory features which identify a particular stimulus. This would fit with the discovery that the earliest wave of activity conveys global category membership, followed by stimulus identity and then more detailed features such as facial expression (Sugase et al. 1999).

The categories of stimuli used in most of these simulations were artificial and highly idealised in several respects. While using natural 3-D stimuli would have been more realistic, the use of abstract stimuli in this way (rather than Gabor-filtered 3-D shapes) allowed the details of the mechanism to be more easily identified without being obscured by additional simulation details. Furthermore, the abstract nature of the stimuli (as distributed representations of input features) allows for a more general interpretation of them than as strictly visual stimuli. In principle, the same mechanism could therefore model the learning processes operating in other sensory modalities where multiple stimuli undergo identity-preserving transformations, (although for clarity we restrict further discussion to the visual domain).

Similarly, translations were chosen as a specific case of identity-preserving transformation as they can be easily demonstrated with abstract sets of stimuli and a simple network architecture. While a direct demonstration of other types of transformation (particularly 3-D transformations such as changes of viewpoint) would have been ideal, the simplicity of the model means that translations may serve as examples of the general case. Unlike other biologically inspired models of object recognition such as HMAX (Riesenhuber and Poggio 1999) and the Neocognitron (Fukushima 1980, 1988), which feature explicit pooling of features across different spatial scales or locations, one set of input features has no special status relative to any others prior to learning. Hence, to the output neurons of the network, there is computationally no difference between associating the same features in a different location (as in translations) or a slightly different set of features in the same location (as in changes of view). In either scenario, there are overlapping sets of input features that must be associated together onto the same output neurons through learning in order to build transformation-invariant representations.

In the presented work, only two stimuli were presented at a time. While this is a significant advance on much of the existing work (where stimuli are presented individually), it still lacks some realism compared to natural visual scenes which may be composed of many more objects. This issue of capacity was previously explored in our earlier work, however (Evans and Stringer 2013), where it was found that approximately eight stimuli could be simultaneously segmented at a biologically plausible frequency of oscillation. Considering that only a small proportion of a visual scene is brought into focus on the fovea at any particular moment, this may be adequate to segment stimuli being actively attended to.

A further limitation of the stimuli used in many of the presented simulations is that there were no features in common between the stimulus categories. In the visual system, however, stimuli may share several features with those from other categories. As demonstrated with the simulations using overlapping categories, if these shared features are regularly present in examples of each category and constitute a large proportion of the visible features, they will tend to bring the stimuli into synchrony with one another.

However, during early visual experience, the statistical properties of the features would naturally form categories according to the frequency with which they are associated. Consequently, the more prototypical features of a category would be most strongly associated together, while less characteristic features (also found in members of other categories) would be relatively weakly connected. The connections of these shared features therefore may not be strong enough to synchronise two example stimuli in the presence of the more canonical features. Furthermore, with more natural stimuli, other mechanisms of differentiation may be available, such as spatial separation or independent movement, which have been shown to help segment a visual scene (Tromans et al. 2012; Evans and Stringer 2013). This would be interesting to explore in a more detailed model using filtered natural images as stimuli.

The same processes demonstrated here to encode category identity in lateral connections may be viewed from another perspective. If we consider the visual world to be more commonly composed of continuous contours rather than abrupt changes in feature orientation, low level visual features with similar orientation preferences would be more commonly coactive than those sets with perpendicular orientation preferences. Such statistics, combined with the lateral plasticity as described, would naturally lead to lateral connections between similar receptive field (orientation) preferences, as are found in the visual cortex of several species (Malach et al. 1994), potentially providing a neurophysiological explanation for the Gestalt principle of ‘good continuation’ or colinearity.

In future work, this mechanism could be applied to model the emergence of other Gestalt principles of perception. For example, if the connections between visual motion sensitive cells are self-organised in the same way, we expect that cells representing similar motion vectors would associate across the visual field, providing a basis for the principle of ‘Common fate’. In a more complex model with Gabor-filtered realistic visual inputs, such learned connections could also help to segment partially occluded stimuli, for example, based upon their texture or continuation of edges, and would provide an explanation for perceptual effects such as illusory contours.

## References

[CR1] Bi G-Q, Poo M-M (1998). Synaptic modifications in cultured hippocampal neurons: dependence on spike timing, synaptic strength, and postsynaptic cell type. J Neurosci.

[CR2] Bishop CM (1997). Neural networks for pattern recognition.

[CR3] Connor CE, Brincat SL, Pasupathy A (2007). Transformation of shape information in the ventral pathway. Curr Opin Neurobiol.

[CR4] DiCarlo J, Zoccolan D, Rust N (2012). How does the brain solve visual object recognition?. Neuron.

[CR5] DiCarlo JJ, Cox DD (2007). Untangling invariant object recognition. Trends Cogn Sci.

[CR6] Elliffe MCM, Rolls ET, Stringer SM (2002) Invariant recognition of feature combinations in the visual system. Biol Cybern 86(1):59–71 10.1007/s004220100284 11924570

[CR7] Engel AK, König P, Singer W (1991). Direct physiological evidence for scene segmentation by temporal coding. Proc Natl Acad Sci.

[CR8] Evans BD, Stringer SM (2012). Transformation-invariant visual representations in self-organizing spiking neural networks. Front Comput Neurosci.

[CR9] Evans BD, Stringer SM (2013). How lateral connections and spiking dynamics may separate multiple objects moving together. PLoS ONE.

[CR10] Felleman DJ, Van Essen DC (1991). Distributed hierarchical processing in the primate cerebral cortex. Cereb Cortex.

[CR11] Földiák P (1991). Learning invariance from transformation sequences. Neural Comput.

[CR12] Földiák P, Eeckman FH, Bower JM (1993). The ‘Ideal Homunculus’: statistical inference from neuronal population responses. Computation and neural systems, Chapter 9.

[CR13] Freeman J, Simoncelli EP (2011). Metamers of the ventral stream. Nat Neurosci.

[CR14] Fukushima K (1980). Neocognitron: a self-organizing neural network model for a mechanism of pattern recognition unaffected by shift in position. Biol Cybern.

[CR15] Fukushima K (1988). Neocognitron: a hierarchical neural network capable of visual pattern recognition. Neural Netw.

[CR16] Gray CM, König P, Engel AK, Singer W (1989) Oscillatory responses in cat visual cortex exhibit inter-columnar synchronization which reflects global stimulus properties. Nature 338(6213):334–337 10.1038/338334a0 2922061

[CR17] Gütig R, Aharonov R, Rotter S, Sompolinsky H (2003). Learning input correlations through nonlinear temporally asymmetric Hebbian plasticity. J Neurosci.

[CR18] Hung CP, Kreiman G, Poggio T, DiCarlo JJ (2005). Fast readout of object identity from macaque inferior temporal cortex. Science.

[CR19] Ito M, Tamura H, Fujita I, Tanaka K (1995). Size and position invariance of neuronal response in monkey inferotemporal cortex. J Neurophysiol.

[CR20] Kobatake E, Tanaka K (1994). Neuronal selectivities to complex object features in the ventral visual pathway of the macaque cerebral cortex. J Neurophysiol.

[CR21] Li N, DiCarlo JJ (2008). Unsupervised natural experience rapidly alters invariant object representation in visual cortex. Science.

[CR22] Liu Y-H, Wang X-J (2001). Spike-frequency adaptation of a generalized leaky integrate-and-fire model neuron. J Comput Neurosci.

[CR23] MacKay DJC (2003). Information theory, inference & learning algorithms.

[CR24] Malach R, Tootell RBH, Malonek D (1994). Relationship between orientation domains, cytochrome oxidase stripes, and intrinsic horizontal connections in squirrel monkey area V2. Cereb Cortex.

[CR25] Masquelier T, Hugues E, Deco G, Thorpe SJ (2009). Oscillations, phase-of-firing coding, and spike timing-dependent plasticity: an efficient learning scheme. J Neurosci.

[CR26] Masquelier T, Thorpe SJ (2007). Unsupervised learning of visual features through spike timing dependent plasticity. PLoS Comput Biol.

[CR27] McCormick DA, Connors BW, Lighthall JW, Prince DA (1985). Comparative electrophysiology of pyramidal and sparsely spiny stellate neurons of the neocortex. J Neurophysiol.

[CR28] Miconi T, VanRullen R (2010). The gamma slideshow: object-based perceptual cycles in a model of the visual cortex. Front Hum Neurosci.

[CR29] Milner PM (1974). A model for visual shape recognition. Psychol Rev.

[CR30] Nischwitz A, Glünder H (1995). Local lateral inhibition: a key to spike synchronization?. Biol Cybern.

[CR31] Op de Beeck H, Vogels R (2000). Spatial sensitivity of macaque inferior temporal neurons. J Comp Neurol.

[CR32] Panzeri S, Treves A (1996). Analytical estimates of limited sampling biases in different information measures. Netw Comput Neural Syst.

[CR33] Perrinet L, Delorme A, Samuelides M, Thorpe SJ (2001). Networks of integrate-and-fire neuron using rank order coding A: how to implement spike time dependent Hebbian plasticity. Neurocomputing.

[CR34] Perry G, Rolls ET, Stringer SM (2006). Spatial vs. temporal continuity in view invariant visual object recognition learning. Vis Res.

[CR35] Riesenhuber M, Poggio T (1999). Hierarchical models of object recognition in cortex. Nat Neurosci.

[CR36] Rolls ET, Deco G (2002). Computational neuroscience of vision.

[CR37] Rolls ET, Milward T (2000). A model of invariant object recognition in the visual system: Learning rules, activation functions, lateral inhibition, and information-based performance measures. Neural Comput.

[CR38] Rolls ET, Treves A, Tovee MJ (1997). The representational capacity of the distributed encoding of information provided by populations of neurons in primate temporal visual cortex. Exp Brain Res.

[CR39] Rousselet GA, Thorpe SJ, Fabre-Thorpe M (2004). How parallel is visual processing in the ventral pathway?. Trends Cogn Sci.

[CR40] Song S, Miller KD, Abbott LF (2000). Competitive Hebbian learning through spike-timing-dependent synaptic plasticity. Nat Neurosci.

[CR41] Spratling MW (2005) Learning viewpoint invariant perceptual representations from cluttered images. IEEE Trans Pattern Anal Mach Intell 27(5):753–761 10.1109/TPAMI.2005.105 15875796

[CR42] Stringer SM, Perry G, Rolls ET, Proske JH (2006) Learning invariant object recognition in the visual system with continuous transformations. Biol Cybern 94(2):128–142 10.1007/s00422-005-0030-z 16369795

[CR43] Stringer SM, Rolls ET (2008). Learning transform invariant object recognition in the visual system with multiple stimuli present during training. Neural Netw.

[CR44] Stringer SM, Rolls ET, Tromans JM (2007). Invariant object recognition with trace learning and multiple stimuli present during training. Netw Comput Neural Syst.

[CR45] Sugase Y, Yamane S, Ueno S, Kawano K (1999) Global and fine information coded by single neurons in the temporal visual cortex. Nature 400(6747):869–873 10.1038/23703 10476965

[CR46] Tanaka K (1996). Representation of visual features of objects in the inferotemporal cortex. Neural Netw.

[CR47] Tovée MJ, Rolls ET, Azzopardi P (1994). Translation invariance in the responses to faces of single neurons in the temporal visual cortical areas of the alert macaque. J Neurophysiol.

[CR48] Treves A, Panzeri S (1995). The upward bias in measures of information derived from limited data samples. Neural Comput.

[CR49] Tromans JM, Page H, Stringer SM (2012). Learning separate visual representations of independently rotating objects. Netw Comput Neural Syst.

[CR50] Troyer TW, Krukowski AE, Priebe NJ, Miller KD (1998). Contrast-invariant orientation tuning in cat visual cortex: thalamocortical input tuning and correlation-based intracortical connectivity. J Neurosci.

[CR51] Usher M, Donnelly N (1998). Visual synchrony affects binding and segmentation in perception. Nature.

[CR52] van Rossum MCW, Bi G-Q, Turrigiano GG (2000). Stable hebbian learning from spike timing-dependent plasticity. J Neurosci.

[CR53] VanRullen R, Thorpe SJ (2002) Surfing a spike wave down the ventral stream. Vis Res 42(23):2593–2615 10.1016/s0042-6989(02)00298-5 12446033

[CR54] von der Malsburg C (1999). The what and why of binding: the modeler’s perspective. Neuron.

[CR55] Wallis G, Rolls ET (1997). Invariant face and object recognition in the visual system. Prog Neurobiol.

